# Efficacy of different treatment regimes against setariosis (*Setaria tundra*, Nematoda: Filarioidea) and associated peritonitis in reindeer

**DOI:** 10.1186/1751-0147-50-49

**Published:** 2008-12-16

**Authors:** Sauli Laaksonen, Antti Oksanen, Toomas Orro, Harri Norberg, Mauri Nieminen, Antti Sukura

**Affiliations:** 1 Finnish Food Safety Authority Evira, Fish and Wildlife Health Research Unit, PO Box 517, FI-90101 Oulu, Finland; 2 Department of Animal Health and Environment, Estonian University of Life Sciences, Kreutzwaldi 62, 51014 Tartu, Estonia; 3 Finnish Game and Fisheries Research Institute, Reindeer Research Station, Toivoniementie 246, FI-99910 Kaamanen, Finland; 4 Department of Basic Veterinary Sciences, Faculty of Veterinary Medicine (FINPAR), University of Helsinki, P.O. Box 66, FI-00014 Helsinki, Finland

## Abstract

**Background:**

When a severe peritonitis outbreak in semi-domesticated reindeer was noticed in 2003 in Finland, the concerned industry urged immediate preventive actions in order to avoid detrimental effects of *S. tundra *and further economical losses. A research programme was swiftly initiated to study *S. tundra *and its impact on the health and wellbeing of reindeer.

**Methods:**

The ultimate aim of this study was to test the efficacy of different treatment regimes against *S. tundra *and associated peritonitis in reindeer. The timing of the trials was planned to be compatible with the annual rhythm of the reindeer management; (1) the treatment of calves in midsummer, during routine calf ear marking, with ivermectin injection prophylaxis and deltamethrin pour-on solution as a repellent against insect vectors, (2) the treatment of infected calves in early autumn with ivermectin injection, and (3) ivermectin treatment of breeding reindeer in winter. The results were assessed using the post mortem inspection data and *S. tundra *detection. Finally, to evaluate on the population level the influence of the annual (late autumn-winter) ivermectin treatment of breeding reindeer on the transmission dynamics of *S. tundra*, a questionnaire survey was conducted.

**Results:**

In autumn, ivermectin treatment was efficient against peritonitis and in midsummer had a slight negative impact on the degree of peritonitis and positive on the fat layer, but deltamethrin had none. Ivermectin was efficient against adult *S. tundra *and its smf. All the reindeer herding cooperatives answered the questionnaire and it appeared that antiparasitic treatment of reindeer population was intense during the study period, when 64–90% of the animals were treated. In the southern part of the Finnish reindeer husbandry area, oral administration of ivermectin was commonly used.

**Conclusion:**

Autumn, and to a lesser degree summer, treatment of reindeer calves with injectable ivermectin resulted in decreased severity of peritonitis and perihepatitis in reindeer calves due to setariosis. In the case of necessity for animal welfare reasons, treatment during early autumn round ups should be considered. On the population level, massive and routinely applied antiparasitic treatments can improve the health of breeding reindeer and decrease the mortality and the number of carriers but during the outbreak could not prevent its movement and expansion to the North.

## Background

In 2003, an emerging peritonitis outbreak caused by *Setaria tundra *appeared in reindeer (*Rangifer tarandus tarandus*) in Finland. The proportion of reindeer calf viscerae condemned due to lesions possibly associated with *S. tundra *in meat inspection increased from 4.9% in 2001 to 40.1% in 2003 in the southern part of the Finnish reindeer herding area and subsequently the focus of the outbreak moved approximately 200 km northwards during next two years. [1]

The genus *Setaria *(Filarioidea: Onchocercidae) includes 43 species that are found mostly in the abdominal cavities of artiodactyls, but also in equids and hyracoids [2]. All produce microfilariae (mf) which are present and available for vectors in host blood. Known vectors are haematophagous mosquitoes (Culicidae spp) [2] and horn flies (*Haematobia *spp.) [3].

*Setaria tundra *was first described in semi-domesticated reindeer in Arkhangelsk area [4], and later reported in reindeer from the Baikal area [5]. Peritonitis caused by *S. tundra *was first in 1973 seen in Swedish reindeer [6]. Further, in 1973, *S. tundra *was observed for the first time in northern Norway, also there associated with an outbreak of peritonitis [7]. Moreover in 1973, tens of thousands of reindeer died in the northern part of the Finnish reindeer husbandry area. Following this, the parasite became oblivious in reindeer in Finland until the outbreak emerged again in 2003 [1].

Endectocidic antiparasitic treatment is widely and routinely used in Finnish reindeer management in every late autumn and early winter when the breeding reindeer are treated with ivermectin during autumn round-ups or later in winter corrals in areas where they are gathered for feeding for winter months. Originally, treatment with various insecticides was targeted against warbles (*Hypoderma tarandi*) and throat bots (*Cephenemyia trompe*). After the discovery of ivermectin, the first endectocidic macrocyclic lactone, the spectrum of the routine antiparasitic treatment broadened to control also potentially harmful gastrointestinal nematodes [8]. To our knowledge, there are no earlier reports on the effect of ivermectin against *S. tundra*.

Haematophagus insects are supposed to serve as vectors of *S. tundra *and the principal transmission time is during the mass appearance of insects (räkkä-time) coexistent with or following the microfilaremia peak in reindeer blood circulation, from middle of June to the end of July [9].

Insecticides have played a central role in controlling the major insect vectors of infectious diseases such as malaria, filariosis and haemorrhagic fever since the early 20th century. Deltamethrin is a synthetic pyrethroid with a strong insecticidic effect and a good molecular stability against adverse environmental conditions such as sunshine and rainfall, as reviewed by [10]. Pyrethroids are known to possess high activity against a broad spectrum of insect pests, both adults and larvae, with low acute toxicity against mammals and lack of persistence in the environment [11-13]. Because of these properties deltamethrin is used widely also in northern Finland, for controlling lice and flies inside cow barns and for controlling mosquitoes and biting flies on pastures.

In Finland, reindeer husbandry is practiced by a system of reindeer herding cooperatives. They are geographically defined profit-making reindeer husbandry units whose members, or shareholders, are reindeer owners. When the peritonitis outbreak in semi-domesticated reindeer was first noticed in 2003 in the south eastern part of the Finnish reindeer-herding area, the Reindeer Herders' Association and individual cooperatives urged immediate antiparasitic treatment trials in order to prevent detrimental effects of *S. tundra *and further economical losses.

A research programme was swiftly initiated to study *S. tundra *and its impact on the health and wellbeing of reindeer. The timing of the field trials was planned to follow the natural annual rhythm of the reindeer management and in periods when the mostly free ranging reindeer could be handled. The reindeer are driven to large herds in Midsummer by the plague of blood-sucking insects and in autumn by rutting season. In these periods the reindeer are rounded up into summer, autumn or winter corrals for various tasks such as counting, ear marking, transportation and slaughter.

The ultimate aim of the study was to test the efficacy of different treatment regimes against *S. tundra *and associated peritonitis in reindeer. On the individual level we aimed to learn if the treatments could prevent or decrease the infection of reindeer calves, decrease the pathology leading to carcass condemnation and improve health of calves. On the population level the aim was to evaluate the influence of the annual (late autumn-winter) ivermectin treatment of breeding reindeer on the population dynamics and the transmission of *S. tundra *from carriers to calves. To achieve these goals, we conducted three field trials at three different times of the year: (1) the treatment of calves in midsummer, during routine calf ear marking, with ivermectin injection prophylaxis and deltamethrin pour-on solution as a repellent against insect vectors, (2) the treatment of infected calves in early autumn with ivermectin injection, and (3) ivermectin treatment of breeding reindeer in winter. Finally a questionnaire survey was conducted including a collection of the status of the antiparasitic treatment in the Finnish reindeer management during the outbreak.

## Materials and methods

**Figure 1:**
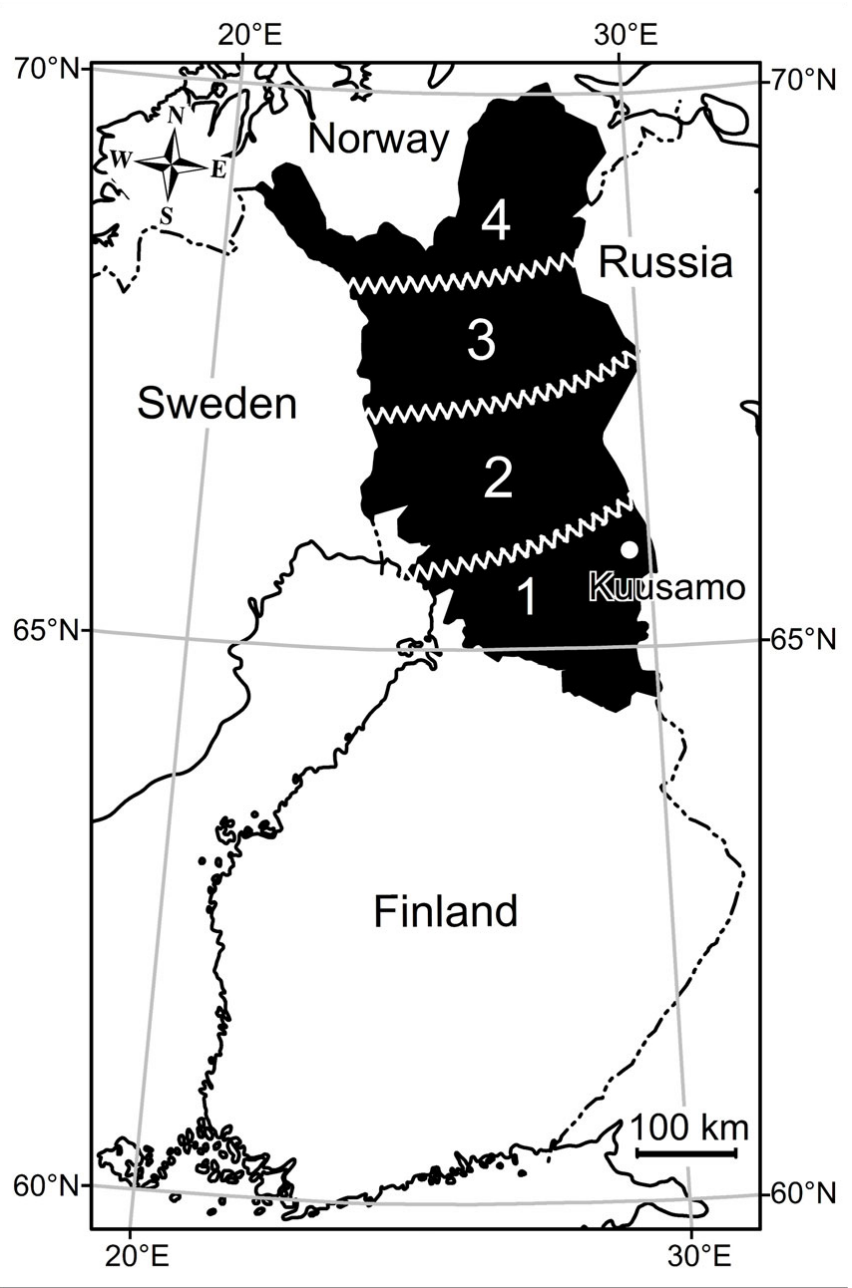
The Finnish reindeer husbandry area (black) divided into four areas to estimate the efficiency of the yearly ivermectin treatment against *S. tundra *on the population level.

The experiments were made in Kuusamo, a then highly *S. tundra *endemic area in the south-eastern part of the Finnish reindeer husbandry area (Fig 1). In 2003, the prevalence of *S. tundra *infection in the area was 89% in calves and 36% in adults, and the corresponding peritonitis prevalence in calves was 57% [1]. All the post mortem inspections were performed in Kuusamo reindeer slaughterhouse during routine slaughter. The post mortem inspections and meat inspection decisions were made by an experienced reindeer meat inspection veterinarian (S. Laaksonen).

### Autumn ivermectin trial (Trial I)

On November 11^th^, 2003, twenty two random reindeer calves were captured during autumn slaughter round-up. Eleven calves (7 males, 4 females) were treated with ivermectin injection (estimated body weight 50 kg; 200 μg/kg b.w. s.c. Virbamec^® ^10 mg/ml, Virbac), and another eleven (8 males, 3 females) formed the untreated control group. Both groups were individually marked with number ear tags and released back to range free until slaughter. Six calves from both groups were slaughtered on December 16^th ^(35 days post treatment), and the rest on January 12^th^, 2004 (62 days post treatment), for post mortem inspection, *S. tundra *detection in abdominal cavity and mf detection in the blood. The degree of peritonitis and perihepatitis was estimated according to [1] and *S. tundra *microfilaria (smf) detection was made from blood as described earlier in [9].

### Winter ivermectin trial (Trial II)

Another experiment was carried out just after reindeer slaughter season 2003, after breeding reindeer had been collected to the winter corrals for routine winter management including antiparasitic treatment and (supplemental) feeding. This is the standard treatment regime in Finnish reindeer herding, when the majority of breeding reindeer are adults whereas. Reindeer (n = 200) belonging to six different reindeer owners were allocated into six different corrals according to ownership. All corrals were situated within a 30 km range in the Kuusamo district. In summer the reindeer had been grazing on common summer pastures and in the winter similar supplemental feeding was utilized. The feed included silage, commercial reindeer fodder, dry hay and a small amount of lichen. Initially, reindeer had already been in corrals, from one week to one month prior to the study beginning. The reindeer were randomly divided into two groups, 100 in each, and handled on January18^th^, 2004. All adult reindeer were breeding females while the sex of the calves was not recorded. The first group (22 calves, 78 adults) received ivermectin injection (Bimectin^®^, Vetpharma AB) s.c. in the neck (200 μg/kg b.w). The second group (20 calves, 80 adults) acted as an untreated control group. Blood samples were collected on March 2^nd ^and 3^rd^, 2004 from vena jugularis (Venoject^® ^VP-100SDK) employing 12G Venoject^® ^needles. Subsequently, smf-detection and identification was performed as described by Laaksonen et al. (2008) [9].

### Summer ivermectin and deltamethrin trial (Trial III)

*Setaria tundra *prophylaxis in reindeer calves was tested by giving ivermectin injection as an anthelmintic or deltamethrin pour-on solution as an insect vector repellent in midsummer 2004. The treatment was performed during the calf ear-marking, which took place at the onset of the mass appearance of insect vectors. Calves were treated between June 26^th ^and July 4^th^, 2004. The calves were systematically allocated into groups, 175 animals each, weighed and ear-tagged during the routine earmarking event. One group was treated with ivermectin (Virbamec^®^) injection (200 μg/kg b.w. s.c.) by tuberculine syringes and needles (Terumo, 20G, 1.5 inch), the second group received deltamethrin 50 mg (5 ml Coopersect spot on^® ^10 mg/ml (Schering-Plough Animal Health), on the skin, on the withers by a 5 mm thick plastic catheter. The third group was an untreated control group.

Altogether 109, 108 and 115 calves were slaughtered from the ivermectin group, the deltamethrin group and the control group, respectively. The slaughter of the calves extended from November 4^th^, 2004 until February 7^th^, 2005 in seven slaughter batches. The rest of the marked calves (66, 67 and 60, respectively) were either not observed in the winter round-ups or were left alive for breeding purposes. Those calves not observed at all had most probably succumbed during summer pasturing (predation by large carnivores is high in the area [14]). Post mortem examination was performed on the slaughtered animals and pathological changes were documented.

Blood samples for mf detection were collected and smf recognized and counted as described in [9]. Slaughter weights and back (rump) fat layers were measured by slide calibre, adult *S. tundra *worms were counted and the degree of peritonitis and/or perihepatitis was assessed as described before [1]. The daily weight gain was calculated by subtracting the live weight at marking from live weight at slaughter and dividing the result with days between marking and slaughter. The live weight at slaughter was estimated by the relationship of carcass weight to the body weight, which in reindeer calves is around 50% [15]. To exclude the impact of other parasites on the wellbeing of the calves, faecal samples were collected from the rectum and examined fresh for the eggs of Trichostrogylidae spp. *Nematodirus *spp., *Capillaria *sp. and *Moniezia *sp. and oocysts of *Eimeria *spp. Faecal samples were examined according to a modified McMaster method, each egg representing 20 eggs per gram (epg) at Evira Fish and Wildlife Health Research Unit laboratory in Oulu. Warble fly larvae were counted during skinning at slaughter.

### Questionnaire

The status of the antiparasitic treatment (prevalence of treatment, proportions of injection and oral treatment) in all 56 cooperatives of the Finnish reindeer herding area in 2002, 2003 and 2004 was collected by a questionnaire addressed to the chiefs of the cooperatives. The questionnaire was performed by a form which was delivered by post and in some cases completed by phone call.

The reindeer-herding area was divided into four areas as described to estimate the efficiency of the yearly ivermectin treatment against *S. tundra *in the population level (Fig. 1) (see also [9]).

### Statistic

Statistical analyses were performed with Stata 9 (StataCorp LP, USA) software.

In the first and second trial the Pearson' chi square test was used to analyse ivermectin treatment effect to the meat inspection findings and to the *S. tundra *and smf prevalence.

In trial three, logistic regression was used to analyse the medication effect (two medications versus no treatment) to the degree of peritonitis (0–1 versus 2–3 in [1]), presence of perihepatitis, prevalence of *S. tundra *nematodes and smf in blood or to the prevalence of other parasites. Logistic regression was also used for studying associations between peritonitis degree and perihepatitis with the prevalence of nematodes in abdominal cavity or smf in blood. Pearson goodness-of-fit test was used to validate all logistic regression models. Differences between treatment groups and controls in slaughter weight, daily weight gain and thickness of back fat layer were tested using linear regression models. Days from treatment to slaughter were included in all linear regression models as significant predictor. Sex was included in daily weight gain and slaughter weight models and weight at treatment in daily weight gain model. Model assumptions were checked using scatter and normality plots of the models' standardised residuals. Square root transformation of fat layer was used to achieve validity of linear regression model. The level of significance was set at 5% (p < 0.05).

## Results

### Trial I

In post mortem examination during the slaughter, none of the ivermectin group calves were found to have live *S. tundra *nematodes in abdominal cavity whereas all the reindeer in the control group had an average of 4.5 (SD 6.25) live worms (p < 0.001). In eight calves (73%) of the ivermectin group, dead, more or less degenerated nematodes or their remains were detected in contrast to the control group where no dead nematodes were found. All the reindeer (n = 11) in the control group and three reindeer (27%) of the ivermectin group slaughtered in the latter slaughter batch on January 12^th^, 2004, had smf in the blood circulation (p < 0.001). There was a significant (p < 0.001) difference in prevalence of peritonitis (peritonitis degrees 0–1 versus 2–3) between groups. Nine of the calves in the control group had peritonitis degree 2–3 and only one in the ivermectin group. In all the six animals with peritonitis (five degree one, one degree 2) in the ivermectin group, the peritonea were described as being dry and having organized lesions whereas peritonea of all the animals in the control group were wet with 50 to 150 ml of ascites fluid in the abdomen.

### Trial II

The calf/adult ratio was not different in the ivermectin and control groups (p = 0.728). The prevalence of smf in the ivermectin group was nine times smaller than in the control group. The results are presented in Table 1.

**Table 1:** Number of reindeer and the prevalence of *S. tundra *microfilariae in the ivermectin and control groups 44 days after ivermectin injections (200 μg/kg b.w. s.c.) in winter ivermectin trial.

**Group**	**Ivermectin**	**Control**	**p-value (chi)**
Calves (n) Smf prev.	22 **14%**	20 **95%**	p < 0.001
Adults (n) Smf prev.	78 **1%**	80 **34%**	p < 0.001
Total (n) Smf prev.	100 **4%**	100 **36%**	p < 0.001

### Trial III

The results obtained at slaughter are presented in Table 2. There was no significant impact of antiparasitic treatment in the degree of peritonitis (degrees 0–1 versus higher than degree 1), prevalence of *S. tundra *nematodes in the abdominal cavity or smf in blood. However, significantly less animals with perihepatitis (p = 0.018) were found in the ivermectin group compared with the control group. The degree of peritonitis was positively associated with finding of *S. tundra *nematodes in the abdomen (p = 0.002) and smf in the blood (p = 0.047). The prevalence of parasites, both adult *S. tundra *(p < 0.001) and smf in the blood circulation (p = 0.003) and also the degree of peritonitis (p < 0.001) were positively associated with the prevalence of perihepatitis.

**Table 2:** Number of reindeer calves, and weight, health and parasitological parameters assessed during slaughter after ivermectin or deltamethrin treatment during ear marking in midsummer.

**Group** n (male/female)	**Ivermectin** 109 (63/46)	**Deltamethrin** 108 (60/48)	**Control** 115 (64/51)
Weight during treatment mean, SD	19,3 kg, 4.6	20.8 kg, 3.8	21 kg, 3.7
Carcass weight at slaughter mean, SD	23,4 kg, 3.2	23.8 kg, 2.7	23.7 kg, 3.0
Weight gain mean, SD	27.6 kg, 5.7	26.6 kg, 4.8	26.5 kg, 5.4
Daily weight gain mean, SD	194 g, 0.6	185 g, 0.5	185 g, 0.5
Fat layer mean, SD	4.7 mm, 4.9	4.1 mm, 3.2	3.7 mm, 4.2
Peritonitis (degree 2–3)	8%	20%	16%
Perihepatitis (degree 1–3)	48%^1^	66%	64%
*S. tundra *in abdomen	69%	69%	71%
Smf in blood	23%	22%	25%
*Nematodirus *spp. prev.% mean epg in infected, (SD)	18% 43, (34)	20% 39, (25)	16% 46, (32)
Trichostrogylidae spp. prev % mean epg in infected, (SD)	49%, 79, (101)	61% 74, (69)	61% 83, (108)
*Moniezia *sp. prev.% mean epg in infected, (SD)	16% 872, (1144)	16% 552, (883)	18% 989, (1295)
*Capillaria *sp. prev.% mean epg in infected, (SD)	10% 38, (31)	9% 47, (35)	8% 28, (10)
*Eimeria *spp. prev.% mean epg in infected, (SD)	54% 17502, (12237)	45% 553, (1192)	48% 732, (1202)
*Hypoderma tarandi *prev.% (mean in infected)	9% 3.3	6% 2.3	9% 2

^1 ^Significantly different (p = 0.018) from control group

There were no treatment effects on the daily weight gain or on the slaughter weight of calves. Treatment effect on the thickness of the fat layer was also not significant. However, difference between ivermectin and control group was close to significance (thicker in ivermectin group; p = 0.052).

There were no significant differences between the prevalences of *S. tundra *or prevalences and densities of other parasites between treatment groups.

In meat inspection, all the livers classified to perihepatitis degree 1–3 (see [1]) and peritonea classified to peritonitis degree 2–3 were condemned. No other pathological changes or disease leading to condemnation was found in any group.

### Questionnaire

The results of the questionnaire addressed to the chiefs of the reindeer herders cooperatives (n = 56, response rate 100%) about the history of antiparasitic treatment of reindeer with ivermectin in the years 2002, 2003 and 2004 are presented in Table 3 and Fig 1.

**Table 3:** Frequency of antiparasitic – treatment of breeding reindeer in autumn/early winter 2002–2004 with ivermectin inj. 200 μg/kg s.c. or paste 200 μg/kg per os and reindeer densities (see fig 1).

year	2002	2003	2004	Reindeer/km^2 ^in summer 2003
area	inject/paste	inject/paste	inject/paste	

1	52%/24%	57%/23%	68%/17%	1.6
2	82%/4%	83%/4%	75%/2%	2.2
3	77%/0.2%	87%/0.3%	90%/0.2%	2.8
4	59%/5%	64%/5%	66%/5%	3.4
tot	69%/7%	73%/7%	75%/5%	2.5

## Discussion

The major conclusion of this study is that autumn, and to a lesser degree summer, treatment with injectable ivermectin resulted in decreased severity of peritonitis and perihepatitis in reindeer calves due to setariosis. The pathological changes caused by *S. tundra *have been associated to the decrease of the body condition of slaughter calves [1]. In trial III, a slight benefit of midsummer ivermectin treatment was detected in the highly endemic area of *S. tundra*. Fat layer was thicker in the ivermectin group and the quality of livers was better, the liver and, especially the liver capsule, being the primary location of changes caused by *S. tundra*.

Antiparasitic treatment of slaughter calves and its benefits during summer round-ups has been frequently discussed among reindeer herders during the *S. tundra *outbreak and has been practised in some individual cooperatives. In one earlier study in Finland, [16] no benefit of this type of treatment to the summer and autumn growth of reindeer calves could be demonstrated. However, in that work the study conditions differed from the present in that there were no observations of *S. tundra *infection or any associated pathological changes [1] in the study area.

According to the trial I, autumn antiparasitic treatment of reindeer calves during heavy outbreak, before slaughter season, could improve results, maybe not in weight gain indicators, but by reducing condemnations during meat inspection. However the economical feasibility of treatment at this time can be questioned. Chemical treatment of calves to be selected for slaughter could damage the organic reputation of reindeer meat in foodstuff market [8]. In Finland, only veterinarians are allowed to give ivermectin injections to reindeer which increases the costs of the antiparasitic treatment and adding the costs from extra round-ups, total costs could easily exceed the benefit. Nevertheless, in situations where a heavy outbreak is evident and the reindeer calves' welfare demands, efficient antiparasitic treatment is available and should be used.

Ivermectin injection (200 μg/kg b.w. s.c) proved to be efficient against adult *S. tundra *nematodes in the abdominal cavities of calves (trial I). It was also efficient against smf in blood circulation, at least on the individual reindeer level. In trial I, there were no live *S. tundra *nematodes in the abdominal cavity in the treated group. In addition, healing processes and the organization of the inflammatory changes in the abdominal cavity had clearly started one month following the ivermectin treatment. This demonstrates that the peritonitis is associated with living *S. tundra *nematodes in abdominal cavity as earlier presented [1]. The results also show that routine antiparasitic treatment, especially of calves which are left alive for breeding purposes, is well funded.

In trial I, none of the six calves slaughtered 30 days after ivermectin treatment had live smf in the blood circulation, but three of the five calves slaughtered 57 days after treatment had. In trial II, the proportion of smf positives was 4% of the treated reindeer 44 days after ivermectin injections. The results may indicate partial efficiency of ivermectin against migrating larvae [17], which might have subsequently matured and started to produce smf. However, this part of *S. tundra *nematode's life cycle is still poorly understood. The possible bias because of reinfection was not possible because of the luck of insects and *S. tundra *nematodes' 3 to 4 month's pre-patent time [9]. The variable efficiency of ivermectin against the developing stages of filarioidea nematodes has been shown earlier by [17]. Another possibility is that the larval output was temporarily suppressed by ivermectin as shown in other filarioidea species reviewed by [18]. Ivermectin is also commonly used in the control of human filariosis because of its potent effect on mf [19]. Although there is currently no formal evidence for the development of resistance to any drug used against filariasis several cases of 'non- or poor responsiveness' to treatment of onchocerciasis with ivermectin have been reported [20]. This is also important to keep in mind, especially after decades of mass ivermectin treatment of Finnish reindeer population [8]. Overall, the results obtained from our trials were comparable to earlier reports in which the variable efficiency of ivermectin against *Setaria *spp. infection has been demonstrated in domestic animals. These results demonstrate that the efficiency of ivermectin (200 μg/kg b.w.) against the circulating *Setaria *microfilariae (smf) is better, varying from 88 to 100%, than against the adult worms (67–84%) [21-26].

Prevention of *S. tundra *transmission by the insecticide and mosquito repellent, deltamethrin, was not successful. It is possible that the effect of pour-on deltamethrin solution against mosquitoes does not last very long in nature in the occasionally rainy conditions in Finland. In addition, the relatively slight preventive effects of both ivermectin and deltamethrin treatment during mid summer round-ups may be due to the transmission dynamics of *S. tundra*. The development of smf after the mosquito's blood meal to the infective stage is temperature dependent and lasts at least about two weeks [2,27-30]. This is congruent to the conclusion [9] that the most active transmission time of *S. tundra *is between early July and late August. In midsummer, the mass appearance of mosquitoes has just started and by the time when these intermediate hosts become active vectors the plasma concentration of ivermectin has decreased in reindeer [31] and deltamethrin is diluted. A more efficient way to prevent transmission in mid summer could be the medication of the older animals in the herd, the reservoir of *S. tundra *especially when the role of wild cervids as reservoirs for *S. tundra *is in North Finland insignificant [9]. A limitation to this would be the fact that handling is difficult at that period because of the fragile growing velvet antlers of adult reindeer.

It has been proposed [16] that reindeer calves pick up gastro-intestinal parasite larvae at an early age in late June or early July. However the infection pressure of the parasites is likely strong also later in the summer, which may explain why there were no distinguishable differences in the prevalence or intensity of other ecto- and endoparasite infections between groups in this study. Another explanation, parasite resistance to ivermectin because of widespread and long standing use of ivermectin, demands further research efforts.

According to the questionnaire antiparasitic treatment with ivermectin was relatively intensive in 2002–2004: about 80% of Finnish reindeer got medication. The use of equine ivermectin paste administered per os was common in southern areas. In the area 1 in 2002, just before the outbreak, 24% of reindeer got paste and only 52% got ivermectin administered by injection. During the course of the outbreak, the proportion of injection increased to 68%. This was mainly due to advice given by the Reindeer Herders' Association and by the reindeer health care programme by Finnish Food Safety Authority Evira. The use of oral administration of ivermectin was not recommended because of the weaker efficiency against gastrointestinal nematodes [32,33], lower achieved plasma concentrations and the danger of emerging drug resistance [31]. It is unclear if the high proportion of reindeer treated by oral administration of ivermectin is connected with the genesis of the outbreak in this area [1] where the reindeer density is only about half of the densities in the two northern areas (Table 3). Although mf are usually very susceptible to ivermectin, the adult filarioids are not [17]. It is reasonable to think that the lower ivermectin concentration in reindeer achieved by oral administration has lesser efficiency against adult *S. tundra *nematodes. In the area 3, the treatment with ivermectin injection was intensive during the study period, 87% of reindeer in 2003 and 90% in 2004, which is almost as high as it could ever realistically get in reindeer management. Even then, the outbreak emerged to the area; 63% of viscera condemned in 2005 and the prevalence of smf positive reindeer calves increased from 16% in 2004 to 60% in 2006. Simultaneously, the outbreak settled down in the southern area [1,9].

The results obtained from this study give additional evidence of the pathogenity of *S. tundra *to reindeer and suggest that a heavy infection rate may have detrimental effects on *Rangifer *populations. In addition, recent studies give rise to the hypothesis that the currently high prevalence of filarioid nematodes in cervids [1,9] and in cattle [34] in Finland may be associated with the ongoing climate change.

Observations made in this study support the conclusions of the very efficient transmission and potent vectors of *S. tundra *nematodes as well as deductions about the impact of growing immunity against *S. tundra *[9] in regulating the infection degree and dynamics in reindeer populations. This was also seen earlier in an experiment when hinds in the zoo with very low smf value managed to transfer the infection to their offspring and all infected calves cleared the infection within two years [9]. It is likely that on the population level, these factors are more important than the field efficiency of ivermectin in regulating *S. tundra *infection transmission to the next generation. Therefore, it is essential to gain knowledge of the basic biology and dynamics as well as preventive measures, including drug resistance studies, against these nematodes, there is growing interest in the subarctic areas.

## Conclusion

The major conclusion of this study is that autumn, and to a lesser degree summer, treatment of reindeer calves with injectable ivermectin resulted in decreased severity of peritonitis and perihepatitis in reindeer calves due to setariosis. During a heavy outbreak, early autumn treatment can improve the health and the quality of slaughter calves, but it would detract the eminence of reindeer meat. On the other hand, mid-summer treatment of calves during earmarking round-ups with ivermectin or deltamethrin is neither efficient nor cost-effective. Ivermectin has good efficiency against adult *S. tundra *nematodes in abdominal cavity and circulating smf and then there is obligation and good possibility to treat heavily infected reindeer calves with ivermectin injection (200 μg/kg b.w. s.c.) for humane reasons. On the population level, massive and routinely applied antiparasitic treatments can decrease the number of carrier reindeer in next summer. The fact that this could not prevent the movement of the *S. tundra *outbreak to new areas in the North, indicates efficient transmission dynamics of *S. tundra*. On the other hand, the present intensity of antiparasitic treatment may possibly have prevented the mass mortality of breeding reindeer in winter as described earlier in 1973 [1] by improving the health of breeding reindeer.

## Competing interests

The authors declare that they have no competing interests.

## Authors' contributions

SL carried out the design and the realization of the study and trials. SL also drafted the manuscript. AO and AS participated in the design and coordination of the study and were also active in writing process. TO helped and participated in statistical analyzes. HN and MN partly coordinated and designed the study and helped in drafting the manuscript. All authors involved in the analysis of the data, gave their valuable views, read and approved the final manuscript.
